# Modeling liquid rate through wellhead chokes using machine learning techniques

**DOI:** 10.1038/s41598-024-54010-2

**Published:** 2024-03-23

**Authors:** Mohammad-Saber Dabiri, Fahimeh Hadavimoghaddam, Sefatallah Ashoorian, Mahin Schaffie, Abdolhossein Hemmati-Sarapardeh

**Affiliations:** 1 Department of Petroleum Engineering, Shahid Bahonar University of Kerman, Kerman, Iran; 2 Ufa State Petroleum Technological University, Ufa, Russia 450064; 3 Institute of Petroleum Engineering, School of Chemical Engineering, University of Tehran, P.O. Box: 11155-4563, Tehran, Iran; 4 State Key Laboratory of Petroleum Resources and Prospecting, China University of Petroleum (Beijing), Beijing, China

**Keywords:** Wellhead chokes, Machine learning, Choke modeling, Correlation development, Liquid rate of two-phase flow, Adaboost-SVR, Energy science and technology, Engineering

## Abstract

Precise measurement and prediction of the fluid flow rates in production wells are crucial for anticipating the production volume and hydrocarbon recovery and creating a steady and controllable flow regime in such wells. This study suggests two approaches to predict the flow rate through wellhead chokes. The first is a data-driven approach using different methods, namely: Adaptive boosting support vector regression (Adaboost-SVR), multivariate adaptive regression spline (MARS), radial basis function (RBF), and multilayer perceptron (MLP) with three algorithms: Levenberg–Marquardt (LM), bayesian-regularization (BR), and scaled conjugate gradient (SCG). The second is a developed correlation that depends on wellhead pressure (P_wh_), gas-to-liquid ratio (GLR), and choke size (D_c_). A dataset of 565 data points is available for model development. The performance of the two suggested approaches is compared with earlier correlations. Results revealed that the proposed models outperform the existing ones, with the Adaboost-SVR model showing the best performance with an average absolute percent relative error (AAPRE) of 5.15% and a correlation coefficient of 0.9784. Additionally, the results indicated that the developed correlation resulted in better predictions compared to the earlier ones. Furthermore, a sensitivity analysis of the input variable was also investigated in this study and revealed that the choke size variable had the most significant effect, while the P_wh_ and GLR showed a slight effect on the liquid rate. Eventually, the leverage approach showed that only 2.1% of the data points were in the suspicious range.

## Introduction

The momentous attributes of wellhead choke throughout oil and gas production cannot be overemphasized, as it restricts flow to regulate production rate. The adjustment of the production rate is mainly made by the wellhead chokes, which can be minimized by proper management of the production rate, formation damage, and preventing the occurrence of factors such as water and gas coning and sand production^1^. The wellhead chokes can be either fixed (positive) or adjustable, depending on the bean settings. The bean size is fixed with a positive choke, while an adjustable choke is analogous to a variable valve. Due to a pressure drop in the production pipeline and a pressure falling, a bubble point of a two-phase current is created in the chokes. These two-phase components are divided into two categories, critical and subcritical. The critical flow occurs when the velocity of the fluid is higher than the velocity of the sound, and the flow velocity becomes independent of the upstream pressure^2^. Conversely, in subcritical flow, the flow rate depends on the pressure difference, and changes in the upstream pressure affect the downstream pressure^3^. Numerous techniques exist for forecasting choke patterns in these areas, and it is equally important to predict the boundary between critical and subcritical flow. For instance, at critical flow, the pressure downstream of the choke can be as low as 50% or 5% of the pressure upstream of the choke^4^. The major problem created by two-phase flow via chokes is calculating the flow rate based on measurable parameters such as GLR, bean size, pressure, etc. The methods offered for multiphase flow through chokes fall into two categories, analytical and empirical^5^. In 1949, Tangerang et al. made the first theoretical study of two-phase flow limitations. He assumed the polytropic expansion of a gas uniformly distributed in a mixture into its continuous phase with a liquid^6^. Since then, several approaches have been proposed to predict multiphase flow through chokes. These techniques can be classified into several groups. One group involved simple empirical equations similar to those of Gilbert. In 1954, Gilbert proposed an empirical equation for determining the liquid flow rate, in which the flow is linearly proportional to the P_wh_^7^. Later, this equation was modified by Ros^8^, Achong^9^, Baxendell^10^, pilehvari^11^, Mirzaei and Salavati^12^, and Beiranvand et al. The overall form of the Gilbert Equation is as follows:

1 $${Q}_{liq}=a1\frac{{P}_{wh}^{a2}{D}_{64}^{a3}}{{GLR}^{a4}}$$

where Q_liq_ is the liquid rate (STB/D), D_64_ is choke diameter (1/64in), and Pwh and GLR are wellhead pressure (psi) and gas-to-liquid ratio (SCF/STB), respectively. a1, a2, a3, a4, a5, and a6 are the empirical coefficients of this equation presented in Table 1.

**Table 1:** Specific empirical coefficient correlations proposed for liquid flow through oilfield chokes.

Author	Formula	Coefficient
Gilbert^7^	$QL=a1\frac{{P}_{wh}^{a2}{D}_{c}^{a3}}{{GLR}^{a4}}$	a1 = 0.1; a2 = 1; a3 = 1.89; a4 = 0.546
Ros^13^	$QL=a1\frac{{P}_{wh}^{a2}{D}_{c}^{a3}}{{GLR}^{a4}}$	a1 = 0.05747; a2 = 1; a3 = 2; a4 = 0.5
Achong^9^	$QL=a1\frac{{P}_{wh}^{a2}{D}_{c}^{a3}}{{GLR}^{a4}}$	a = 0.26178; a2 = 1; a3 = 1.88; a4 = 0.65
Pilehvari^11^	$QL=a1\frac{{P}_{wh}^{a2}{D}_{c}^{a3}}{{GLR}^{a4}}$	a1 = 0.021427; a2 = 1; a3 = 2.11; a4 = 0.313
Beiranvand et al.^14^	$QL=a1\frac{{P}_{wh}^{a2}{D}_{c}^{a3}}{{GLR}^{a4}}$	a1 = 0.0382; a2 = 1; a3 = 2.275; a4 = 0.589
Al-Attar^16^	$QL=a1\frac{{P}_{wh}^{a2}{D}_{c}^{a3}}{{GLR}^{a4}}$	a1 = 0.016266; a2 = 0.831; a3 = 1.63; a4 = 0.471
Mirzaei-Paiamann et al.^12^	$QL=a1\frac{{P}_{wh}^{a2}{D}_{c}^{a3}{\gamma }_{o}^{a4 }{\gamma }_{g}^{a5}}{{GLR}^{a6}}$	a1 = 0.052439; a2 = 1; a3 = 1.9108; a4 = 0.3988; a5 = 0.1711; a6 = 0.5220
Baxendell^13^	$QL=a1\frac{{P}_{wh}^{a2}{D}_{c}^{a3}}{{GLR}^{a4}}$	a1 = 0.1046; a2 = 1; a3 = 1.93; a4 = 0.546

Following Tangeren, the Ros conducted studies based on the continuous gas phase and extended the Tangeren Eq. (8). Poettmann and Beck improved the Ros equation using 108 production data. They compiled charts for different types of crude oil with varying degrees of API, ranging choke diameter from 4/64 to 28/64 inches and ranging oil flow rates from 10 to 1300 STBD^15^. Al-Attar and Abdul-Majid conducted a study in which they evaluated and compared the available correlations used to assess the performance of multiphase fluid flow through a wellhead choke. They used 155 well-test production datasets from the east Baghdad oilfield^16^. In another study, Abdul-Majid examined correlations developed for predicting liquid rate in oilfield chokes. A dataset including 210 well-test data was used to predict the accuracy of eight correlation models. Additionally, a regression analysis was employed to find correlations that best matched measured data, and as a consequence, four new correlation coefficients were developed. Based on the statistical results, new correlations were more robust than previous ones^17^. Fortunati Presented an empirical equation for both critical and subcritical currents. Additionally, he included a graphical representation and established the demarcation line between critical and subcritical flow^18^. Ashford^19^ and Pilehvari^11^ performed their studies on subcritical currents in the wellhead chokes. They determined the boundary between critical and subcritical flow as a function of fluid properties and GLR. In another study, Al-Attar carried out research work based on the critical flow through the choke. In this study, he used 40 field data based on choke size adjustment and presented a more accurate empirical equation compared to the previous ones^5^. Beiranvand and Babaei Khorzoughi presented an innovative correlation for multiphase flow through surface chokes, integrating recently introduced parameters. They did their research based on 182 production data from one of the Iranian oil fields. They also added temperature, sediment, and water to the Gilbert equation and obtained more confident results than the previous correlations^20^.

Rashid et al. used the collected 276 data and radial basis function-genetic algorithm (RBF-GA) neural network to estimate the flow rate via the wellhead chokes. In this study, the R^2^ values for training and test data were obtained 0.9885 and 0.9795, respectively^21,22^. Mirzaei-paiaman & Salavati using 102 production test data and adding the specific gravity of oil and gas to the general equation of Gilbert reached the following Eq. (12):

2 $${Q}_{L}=\frac{A.{P}_{wh}.{d}^{B}.{\gamma }_{g }^{D}.{\gamma }_{O}^{E}}{{GLR}^{C}}$$

Q_L_, liquid flow rate (STB/D); D_64_, choke size (1/64 inches); P_wh_, wellhead pressure (Psia); Ɣ_o_, oil specific gravity; Ɣ_g_,  gas specific gravity; GLR,  gas to liquid ratio (Scf/STB); and, A, B, C, D, and E are constants.

According to the literature, most of the experimental relationships presented for calculating the flow rate inside the choke can be classified into two categories, linear and non-linear, which typically yield a high error. However, the literature still suffers from the lack of a comprehensive and accurate model for predicting oil flow inside wellhead chokes. Hence, we attempt to develop a new correlation with a lower percentage of error than the empirical relationships presented in the literature. Additionally, we used robust machine learning algorithms to accurately predict liquid rate through the oilfield chokes. To the best of our knowledge, there has been no prior endeavor to undertake this type of modeling.

In this study, the liquid rate in wellhead chokes is modeled using machine learning approaches. To this end, 565 real data points are collected from the literature. Then, for a precise and reliable prediction of oilfield chokes, several ML models of liquid rate are applied. Four kinds of ANNs MLP with three algorithms, RBF, MARS, and Adaboost-SVR, are employed to develop models to accurately predict the liquid rate through the chokes. Furthermore, statistical evaluation and graphical error criteria are used to investigate the validation and reliability of intelligent models and other correlations. In addition, the relative impact of inputs on the liquid rate in wellhead chokes is inspected by applying the relevancy factor definition. Finally, the leverage approach is utilized to investigate the credit and application of the best-proposed model. Therefore, the key contributions of this study can be summarized as follows:

- Gathering a comprehensive dataset of wellhead choke liquid rates, encompassing crucial variables like D_c_, P_wh_, and GLR.
- The development of precise models with minimal errors by employing Adaboost-SVR machine-learning algorithms.
- Developing a new empirical relationship that outperforms the previously developed relationships.
- Conducting sensitivity analysis to identify the relative impact of pressure, choke size, and gas–liquid ratio on the liquid rate in oil field chokes.
- Applying the leverage method to detect anomalous and outlier data associated with liquid rate as reported in the literature.

## Data collection

First, for accurate prediction of the liquid rate of two-phase flow through wellhead chokes, a comprehensive database of 565 data points of liquid rate was collected^12,20, 23–28^. Based on the literature, the most critical elements that affect the choke liquid flow rate are the P_wh_, D_64_, and GLR. As a result, in this study, the liquid flow rate is defined based on the mentioned parameters. The implemented input parameter range and output parameter range are reported in Table 2. Additionally, the input data were analyzed by mean, minimum, maximum, and other parameters, as in Table 3. The liquid rate changes with a minimum value of 205 (STB/Day), a maximum of 25,878 (STB/Day), and an arithmetic 8146.613. The P_wh_ value changes between 50 and 4045 with an arithmetic mean of 1549.699. The statistical dispersion for a liquid rate through chokes was determined by calculating the kurtosis, skewness, and standard deviation, and values of 1.006, 0.760, and 4383.228 were obtained, respectively, which indicates that the data points are spread out over a broader range of values. Skewness is a measure of the level of asymmetry in the distribution of a dataset. Skewness in the normal curve is observed when a data set is asymmetrically distributed. Skewness can be positive, negative, or undefined. Additionally, kurtosis measures the tailedness of the probability distribution of a random variable. Positive kurtosis means that there are several data points in the tail of a distribution, while negative kurtosis results in a few data points in the tail.

**Table 2:** The range of databases used in the developed model.

P_wh_ range (Psia)	GLR range (SCF/STB)	D range (1/64) (in)	Q_liq_ range (STB/Day)	References
261–2935	186–3792	16–64	282–8030	^12^
133–883	36–885	25.6–40	183–9284	^20^
1646–3000	828.1–13,095.1	21–68	668.4–14,480.8	^23^
50–2940	107–3660	24–80	1324–22,150	^24^
60–350	300–1100	16–64	200–3350	^25^
133–881	36–885	25.6–64	205–25,878	^26^
115–4308	158–6100	16–80	198–9643	^28^
1419.7–1827.7	186–272	32–64	3930–17,310	^27^

**Table 3:** Statistical description of the data set used for modeling.

Parameters	P_wh_ (psi)	GLR (SCF/STB)	D (1/64) in	Q_liq_ (STB/Day)
Mean	1594.699	1084.637	53.133	8146.613
Standard Error	39.798	35.917	0.552	184.404
Median	1500.000	915.000	54.000	8000.000
Mode	2400.000	1040.000	64.000	9280.000
Standard Deviation	945.988	853.733	13.117	4383.228
Sample Variance	894,894.137	728,860.845	172.060	19,212,685.885
Kurtosis	− 1.098	6.010	− 0.455	1.006
Skewness	0.222	1.691	− 0.358	0.760
Minimum	50.000	36.000	16.000	205.000
Maximum	4045.000	5706.600	80.000	25,878.000

## Model development

### Multilayer perception neural network (MLPNN)

A neural network processes the data through a learning process, stores it, and makes it available for use. Synaptic weights, connection strengths between neurons, are used to store knowledge^29^. Neural networks which are significantly important in this context, are a powerful, and comprehensive framework for representing non-linear mappings from several input variables to several output variables, where several adjustable parameters govern the form of mapping. Before the emergence of the MLP neural network, in 1958 Frank Rosenblatt invented a neural network called a perceptron^30^. Rosenblatt formed a layer of neurons and called the resulting network a perceptron. However, Rosenblatt's perceptron also had many problems. For instance, it could only solve problems that were linearly separable^31^. In 1969, Minsky and Paper wrote a book called Perceptron. They explored all the perceptron's capabilities and problems in this book. Minsky and Paper proved that the perceptron could only solve problems that are linearly separable^32,33^. Furthermore, the conceptually more appealing neural network model is the MLP model^34,35^. In its most basic form, this model consists of several successive layers. Each layer consists of a small number of units called neurons^36,37^. In this model, the units of each layer are connected to the next layers, which are called links or synapses. A multi-layer perceptron (MLP) comprises a minimum of three layers of nodes: these include an input layer, a hidden layer, and an output layer. MLP employs an administered learning strategy called feedback for training. Its multiple layers and nonlinear activation distinguish MLP from a linear perceptron. If a multilayer perceptron has a linear activation function in all neurons, it maps the weighted inputs of each neuron with this linear function. At that point, utilizing direct polynomial math, it appears that any number related to layers can be decreased to a two-layer input–output model. These functions usually include "Tanh", "Sigmoid", and "Linear". A linear function is typically used for the output layer. These functions are described below^38^:

3 $$Tansig=tanh: h(x)=\frac{{e}^{x}-{e}^{-x}}{{e}^{x}+{e}^{-x}}=\frac{2}{1+{e}^{-2x}}-1$$

4 $$linear=purelin=h(x)=x$$

5 $$sigmoid=logsig: h(x)=\frac{{e}^{x}}{{e}^{x}+1}$$

Consider an MLP with two hidden layers and logsig and tansig activation functions for the two hidden layers and purlin for the output layer, respectively. The output of the model can be calculated by the following formula:

6 $$output=purlin({w}_{3}\times ({\text{log}}sig\left({w}_{2}\times \left(tansig\left({w}_{1}\times x\right)+{b}_{1}\right)\right)+{b}_{2})+{b}_{3}$$

where the bias terms for the 1st and 2nd hidden layers are ${b}_{1}$ and ${b}_{2}$, respectively, and ${b}_{3}$ is the bias of the output layer. In addition, ${w}_{1}$ , ${w}_{2}$, and ${w}_{3}$ are the weight matrixes for the 1st and 2nd, and the output layer, respectively. The activation functions used for the first and second hidden layers are usually tansig and logsig, respectively, in the case of using two hidden layers^38^.

**Figure 1:**
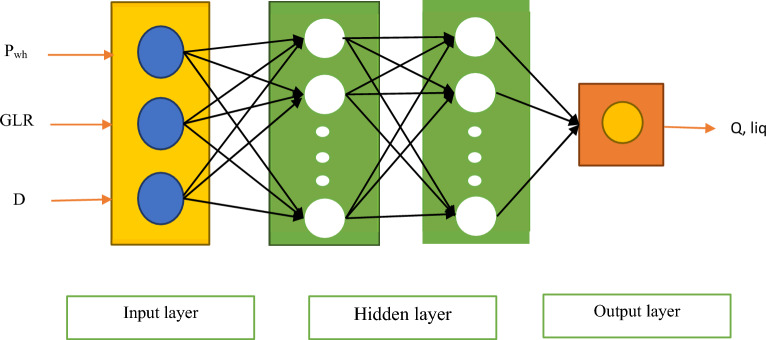
Structural of the MLP model used in this work.

Figure 1 shows the structure of an MLP model with two hidden layers. In this study, to develop the MLP model, three algorithms including Bayesian Regularization (BR), Scaled Conjugate Gradient (SCG), and Levenberg–Marquardt (LM), were used. The type of activation function, the number of neurons, and the number of layers used for the MLP model are reported in Table 4.

**Table 4:** Control parameters for MLP and RBF model used in this study.

MLP	Number of hidden layers	2
	The objective function uses training	MSE
	Optimization algorithm	LM-BR-SCG
RBF	Number of neurons in the hidden layer	250
	Spread	0.1
	The objective function uses training	MSE

### Radial basis function neural network (RBFNN)

**Figure 2:**
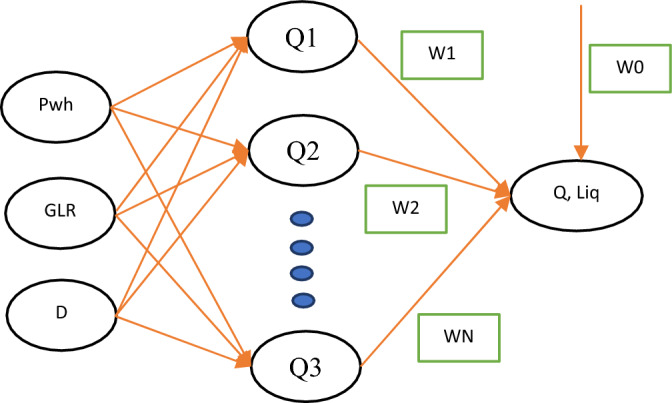
Structural of the RBF model used in this work.

Similar to the MLP neural network model, there is another type of neural network in which processing units are focused on a specific distance. Regarding overall structure, neural RBF networks are not significantly different from MLP networks, and the only difference is the type of processing the neurons perform on their inputs. However, RBF networks often have faster learning and training processes. since neurons are concentrated in specific functional areas, it will be easier to regulate them. Generally, the radial basis function (RBF) network is composed of a three-layer structure, where the initial and final layers serve as the input and output layers, while the intermediate layer functions as the hidden layer. There is one hidden layer in this model that identifies the relationship between input and output data^39,40^. Figure 2 indicates an example of an RBF network. The output of this model is given by the following formula:

7 $${y}_{k}=\sum_{i=1}^{N}{\varnothing }_{ki}\times wi\times (\left|{x}_{i}-{c}_{k}\right|)+{w}_{0} , k=\mathrm{1,2},...,N ;i=\mathrm{1,2},...,M$$

where $wi$, ${w}_{0}$, ${y}_{k}$, $N$, ${c}_{k},$ and $M$ are the weights of the network, the model’s output, the cluster numbers, cluster, coefficient of bias, and data point number, respectively. The maximum number of neurons and the expansion coefficient are the main parameters that can be changed in this model. It should be noted that these factors are usually determined by trial and error.

### Adaptive boosting support vector regression (AdaBoost-SVR)

AdaBoost algorithm is a collective learning method and is a well-known algorithm from the family of Boosting algorithms presented by Freund and Schapire^41^. In collective learning algorithms, one case is classified by several different classifiers, and the classifications’ results are intelligently combined and the final result is determined for that particular case. Typically, the collective learning algorithm is higher compared to the individual classifiers participating in its structure. In AdaBoost collective learning, each class is trained with a different bootstrap. The bootstrap sampling method is such that the number of training samples is randomly selected from the training data set. A nested pattern allows the same pattern to be selected multiple times. This algorithm has several steps that are mentioned here^42^:

1. First, all data will be assigned some weights. Initially, all the weights will be equal. To determine the sample weight, the following formulas were used:
  8 $$w({x}_{i},{y}_{i})=\frac{1}{N} , i=1,2,3,...,n$$
  where N is the total number of data.
2. For m = 1 to M:
  - Fit a classifier G_m_ (x) to the learning data using weights w_i_.
  - Determine
    9 $${err}_{m}=\frac{\sum_{i=1}^{N}{w}_{i}I({y}_{i}\ne {G}_{m}(xi))}{\sum_{i=1}^{N}{w}_{i}}$$
3. Compute
  10 $${\alpha }_{m}=log((1-{err}_{m})/{err}_{m}).$$
4. set $${w}_{i}$$
  11 $$w_{i}^{*} \;exp\left[ {\alpha_{m} .I\left( {y_{i} \ne G_{m} \left( {xi} \right)} \right)} \right]{ },{ }i = 1,2,...,N$$
5. Output
  12 $$G(x)=sign[\sum_{m=1}^{M}{\alpha }_{m}{G}_{m}(x)]$$

where M, ${err}_{m}$, ${\alpha }_{m}$ are the number of learners, the weight of the error rate, and the predicted weight.

### Support vector regression (SVR)

SVR was first proposed in 1995 by Vapnik for classification problems. Recently, the SVR model has become one of the most common models in the field of petroleum engineering due to its acceptable performance in forecasting^43–45^. For a simple case, input data x ϵ R^d^ are regressed by hyper plane g(x):

13 $$g(x)=w.\varnothing (x)+b$$

The weight vector and the bias are w and b, respectively, with g(x) representing the regression function of the input space vector x. A minimization problem is formulated for regression purposes to compute vector b, in which Model complexity and associated empirical error are summarized under the so-called normalized risk function^46^.

14 $$\xi =\left|{y}_{i}-g(w,xi)\right|$$

15 $${\left|\xi \right|}_{\in }= \left\{\begin{array}{l}0\quad \qquad \quad if \left|\xi \right|<\in \\ \left|\xi \right|-\in \quad otherwise \end{array}\right.$$

By considering the positive slack variables ($\xi$, $\xi$*) optimization problem is formulated as:

16 $$\text{Minimize }\frac{1}{2}{\| \omega \| }^{2}+c\sum_{i=1}^{n}({\xi }_{i}+{\xi }_{i}^{*})$$

where $\sum_{i=1}^{n}({\xi }_{i}+{\xi }_{i}^{*})$ represents the empirical error and ${\| \omega \| }^{2}$ is the flatness of the function. C represents a penalizing factor for the data that their deviation from g is higher than ε^47^.

### Multivariate adaptive regression spline (MARS)

MARS is an algorithm designed for multivariate non-linear regression problems^48^. In each aspect, the Mars algorithm divides the input parameter space into separate subregions and corresponds to a spline function known as a basis function. MARS studies non-linear relationships between input and response variables with more flexibility, which is why this model differs from other linear regression techniques. Additionally, MARS checks all degrees of interaction in arrange to discover all conceivable intelligence between factors. This strategy takes into account all intuitive and convenient shapes between input parameters, so it can effectively follow hidden connections in high-dimensional datasets as well as complex structures found in data points^49^. The general formula of this algorithm is represented as follows:

17 $$f(x)={\beta }_{0}+\sum_{m=1}^{m}{\beta }_{m}{\lambda }_{m}(x)$$

where ${\beta }_{0}$ and ${\beta }_{m}$ represent the parameters that give the best fit of data points, f(x) stands for the response, and M indicates BF in the model. In this algorithm, the basis function can take the form of a univariate spline function or a combination of multiple functions, depending on the various predictive inputs. ${\lambda }_{m}$(x) and the spline BF can be presented as follows:

18 $${\lambda }_{m}(x)={\Pi }_{k=1}^{{k}_{m}=1}[{S}_{km}({X}_{v(k,m)}-{t}_{(k,m)}]$$

where ${S}_{km}$ is the right/left regions of the corresponding step function, taking either 1 or − 1, ${t}_{(k,m)}$ represents the knot location, K_m_ presents the number of knots and $v(k,m)$ represents the predictor input’s label. Mars model builds BF using a step-by-step technique. MARS over-fits data in the forward step by investigating an expansive number of BFs. Duplicate BFs are removed backward from the equation to prevent overfitting. To remove duplicate BFs, MARS uses the Generalized Cross-Validation (GCV) criteria. A GCV is expressed as:

19 $$GCV=\frac{\frac{1}{N}\sum_{i=1}^{n}[{y}_{i}-f^({x}_{i})]}{{[1-\frac{C(B)}{N}]}^{2}}$$

The N parameter presents the whole data number. C(B) represents a complexity penalty, and it is defined as^50^:

20 $${\text{C}}\left( {\text{B}} \right) = \left( {{\text{B}} + {1}} \right) + {\text{d}}({\text{B}})$$

### Generalized reduced gradient (GRG)

The generalized reduced gradient (GRG) approach is frequently applied as a solver for multivariable problems. Based on the concept of decreased gradients, this technique is designed to incorporate and solve Linear and non-linear Problems. The component is monitored in such a way as to ensure that the active constraints are kept satisfied when the process changes from one stage to another. The GRG provides a linear estimation of the gradient at a given point x. The constraint and objective gradient are resolved at the same time so that constraints can be represented by gradients of an objective function. By moving in a practical path, the search area is reduced. The following notations represent an objective function, f(z), which is subject to the constraint h(z)^51^.

21 $${\text{Minimizes}}:{\text{ f}}\left( {\text{z}} \right) = {\text{ z}}$$

22 $${\text{Subjected to}}:{\text{ h}}_{{\text{k}}} \left( {\text{z}} \right) = \, 0$$

The GRG can be adjusted using the following form:

23 $$\frac{df}{{dz}_{k}}=\nabla {z}_{k}^{t}f-\nabla {z}_{i}^{t}f\left(\frac{dh}{{dz}_{i}}\right) \frac{dh}{{dz}_{k}}$$

Basically, f(z) will be minimum under two simple conditions which are df(z) = 0 or $\frac{df}{{dz}_{k}}=0$^52^.

## Evaluation of the model

Evaluation of the performance of the proposed models is ordinarily done by comparison of the model prediction with the real values by calculating the various statistical parameters, including average percent relative error (APRE), average absolute percent relative error (AAPRE), standard deviation (SD), root mean square error (RMSE), and coefficient of determination. These statistical parameters are obtained from the following Equations:

24 $$APRE=\frac{1}{n}\sum_{i=1}^{n}Ei$$

where E_i_ is the percent relative error and is stated based on the following formula^53^:

25 $$Ei=[\frac{{\left({Q}_{liq,i}\right)}_{real}-{\left({Q}_{liq,i}\right)}_{pred}}{{\left({Q}_{liq,i}\right)}_{real}}]\times 100$$

26 $$AAPRE=\frac{1}{n}\sum_{i=1}^{n}\left|Ei\right|$$

27 $$SD=\sqrt{\frac{1}{N-1}\sum_{i=1}^{N}{[\frac{{\left({Q}_{liq,i}\right)}_{real}-{\left({Q}_{liq,i}\right)}_{pred}}{{\left({Q}_{liq,i}\right)}_{real}}]}^{2}}$$

28 $$RMSE=\sqrt{\frac{\sum_{i=1}^{N}{\left[{\left({Q}_{liq,i}\right)}_{real}-{\left({Q}_{liq,i}\right)}_{pred}\right]}^{2}}{N}}$$

29 $${R}^{2}=1-\frac{\sum_{i=1}^{N}{\left[{\left({Q}_{liq,i}\right)}_{real}-{\left({Q}_{liq,i}\right)}_{pred}\right]}^{2}}{\sum_{i=1}^{N}{\left[{\left({Q}_{liq,i}\right)}_{real}-\frac{\sum_{i=1}^{N}{\left({Q}_{liq,i}\right)}_{real}}{N}\right]}^{2}}$$

Here ${\left({Q}_{liq,i}\right)}_{real}$ is the real oil flow rate that measured in the field test; ${\left({Q}_{liq,i}\right)}_{pred}$ is the predicted oil flow rate and N presented the whole number of data utilized for analysis.

At the same time, the performance of the machine learning model was assessed using the following graphical tools, which are described further below:

Cross plot: The most widely recognized method is graphical analysis, in which the predicted values are graphed against measured values, and the models' accuracy is determined by how closely the data points align with a line of unity slope.

Cumulative frequency plot: This plot is a comparative chart that can compare several models with each other. In this diagram, a model predicting more data with lower error can be determined. If the model is close to the vertical axis, the higher percentage of data is predicted by a lower error, therefore, it is more accurate than the other model.

Trend plot: This diagram plots both real data and the model's estimate against a given feature or an index to determine whether that model is valid.

Error distribution plot: Plotting the difference between the measured value and the predicted value against the actual data to assess the dispersion of the data around the zero-error line and analyze any patterns in errors.

## Results and discussion

In the present work, models were developed based on 565 production data points that were collected from different sources in the literature. For all models with different algorithms, 80% of the data points were randomly selected to train the set, and the remaining 20% were employed to test and validate the model.

### Development of the correlation

In this work, the GRG algorithm is used to predict the liquid rate through wellhead chokes. The correlation was developed based on four coefficients to optimize the APRE and RMSE, which is presented below:

$$Qliq=a1\times {P}_{wh}^{a2}\times {D}_{c}^{a3}{GLR}^{a4}$$

where Q_liq_, liquid flow rate (STB/Day); P_wh_,  upstream pressure(psi); Dc, choke size (1/64) in and GLR, gas to liquid ratio (SCF/STB).

a1, a2, a3, a4 are equation coefficients are reported in Table 5.

**Table 5:** Coefficients developed correlation to optimized AAPRE and RMSE.

Coefficients	a1	a2	a3	a4
Optimized AAPRE	0.10606	1.10596	1.98196	− 0.70193
Optimized RMSE	0.35152	0.99381	1.82910	− 0.66705

### Statistical analyses of models

First, we have to compare intelligent models and correlation based on statistical parameters including (R2, APRE%, AAPRE %, RMSE, and SD), to find the most accurate and efficient models. Table 6 shows the model development, validation, and statistical evaluation of the total sets for a liquid rate through oil field chokes by Adaboost-SVR, MARS, MLP-LM, MLP-BR, MLP-SCG, and RBF models. Furthermore, Table 7 reports the statistical assessment of the proposed correlations by Gilbert, Ros, Achong, Baxendell, Pilehvari, Beiranvand, and developed correlation to optimized AAPRE and RMSE.

**Table 6:** Statistical evaluation of the developed models.

	Adaboost-SVR	MARS	MLP-LM	MLP-BR	MLP-SCG	RBF
Training set						
AAPRE %	5.3	6.58	8.7	9.24	11.51	8.11
APRE %	− 1.76	− 1.19	− 2.66	− 2.42	− 2.04	− 1.84
RMSE	661.56	469.19	682.99	747.87	917.04	672.42
SD	0.09	0.149	0.31	0.31	0.3	0.22
R^2^	0.9772	0.9889	0.9757	0.9707	0.9525	0.9761
Test set						
AAPRE %	4.57	12.14	9.19	7.92	10.9	8.45
APRE %	− 0.47	− 4.27	− 2.08	− 0.88	− 3.13	1.76
RMSE	564.86	921.01	913.49	710.84	976.23	959.29
SD	0.1	0.31	0.14	0.13	0.19	0.14
R^2^	0.9827	0.9465	0.9571	0.9744	0.9477	0.9559
Total						
AAPRE %	5.15	7.69	8.9	8.74	11.44	8.18
APRE %	− 1.5	− 1.81	− 2.3	− 1.99	− 1.97	− 1.12
RMSE	643.38	588.02	726.34	733.78	958.04	738.76
SD	0.086	0.19	0.29	0.28	0.28	0.2
R^2^	0.9784	0.9819	0.9726	0.9719	0.9522	0.9716

**Table 7:** Statistical analysis errors proposed correlation used in this study and developed correlation.

Model	APRE (%)	AAPRE (%)	RMSE	SD	R^2^
Gilbert	9.84	22.36	2418.76	0.33	0.8118
Ros	− 8.58	21.32	2221.49	0.39	0.7461
Achong	− 14.12	21.05	1641.73	0.43	0.8804
Baxendell	− 10.37	20.36	1915.18	0.40	0.8124
Pilehvari	− 43.76	51.82	5107.94	0.73	0.4230
Al-Attar	32.34	36.97	4206.89	0.43	0.8300
Beiranvand	− 1.54	19.03	1719.97	0.37	0.8502
Developed correlation-optimized AAPRE	− 4.18	17.20	1532.12	0.35	0.8809
Developed correlation-optimized RMSE	− 7.38	18.84	1507.41	0.40	0.8822

As seen in Table 6, using the Adaboost-SVR model results in the lowest value of AAPRE for predicting the liquid rate of two-phase flow through wellhead chokes. The total APRE, AAPRE, RMSE, SD, and R^2^ for Adaboost-SVR are − 1.5%, 5.15%, 643.38, 0.086, and 0.9784, respectively. After Adaboost using the MARS leads to the lowest overall AAPRE. As appeared in this Table, the total AAPRE for MLP-SCG is 11.44% which indicates the lowest precision.

Furthermore, according to the results presented in Table 7, the proposed correlation by Pilehvari has the lowest accuracy compared to other correlations to estimate liquid rate, while using Beiranvand leads to the lowest value of the total AAPRE which is 19.03%. After Beiranvand, using the Achong correlation leads to the lowest value of the overall AAPRE. Comparing the statistical analysis of the errors in Tables 6 and 7, it can be concluded that all the proposed models of ANN had a much higher accuracy than the correlation studied in this research for the prediction of liquid rate in the choke.

To further evaluate the validity and reliability of the Adaboost-SVR model, an external validation dataset containing 28 liquid rates in oilfield chokes over a range of operating choke size (14–48 in), pressure (250–1697.9 psia), and GLR (600.1–800 SCF/STB), were collected from the literature^17^. This data falls entirely outside the training and testing sets utilized for modeling in this paper. As a result, it enables an assessment of the model's performance beyond the data sets used for modeling. Predicted values for Adaboost-SVR are reported in Table 8. The values presented in this Table for experimental and predicted data show that the Adaboost-SVR model demonstrates reliable predictive accuracy even for new fluid rates beyond the range of chokes used during the modeling process.

**Table 8:** The experimental and predicted values for evaluation of the Adaboost-SVR model.

Experimental	Prediction	Experimental	Prediction
5250.7	4207.7	400	400
5220.5	4207.7	500	700
1890.2	1895.2	540	540
1900.2	1900.2	570	770.1
1350.1	1700	690.1	927
6500.8	6758.01	940.1	1158.04
2500.3	2500.3	1100.1	1205.05
1300.1	1300.1	1380.1	1380.1
711	711	1680.1	1532.067
800.1	749.86	1900.5	1680.1
260	330	2050.3	1900.26
290	330	2250.3	2006.325
330	340	3000.4	2919.822
360	360	3500.5	3000.4

### Graphical error analysis

**Figure 3:**
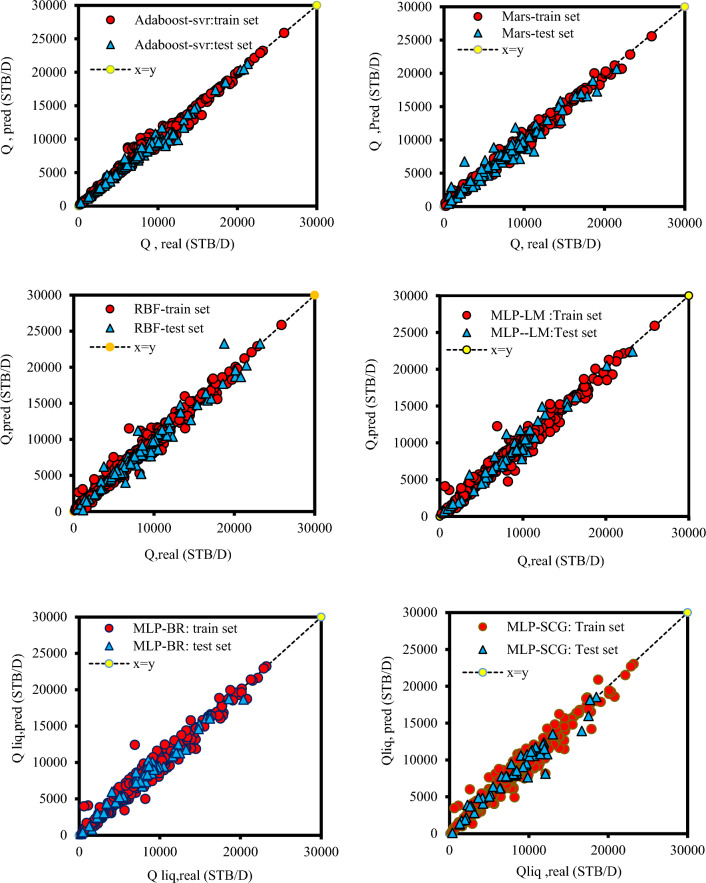
cross-plot for the intelligence models to estimate the liquid rate.

Another way to assess model performance and compare it to other models and proposed correlations is to use graphical error analysis. This graphical strategy impressively helps when there are several models whose performance should be compared together. To assess the precision of the intelligent models consisting of Adaboost-SVR, MARS, MLP-LM, MLP-BR, MLP-SCG, and RBF the predicted liquid rate data was plotted against the real values in Fig. 3. It can be concluded that all intelligence models show relatively good accuracy. The Adaboost-SVR model gives the most noteworthy exactness level compared to other models. Also, it can be concluded that from the Figure MLP with algorithm SCG shows the lowest accuracy compared to the two algorithms MLP-LM and MLP-BR.

**Figure 4:**
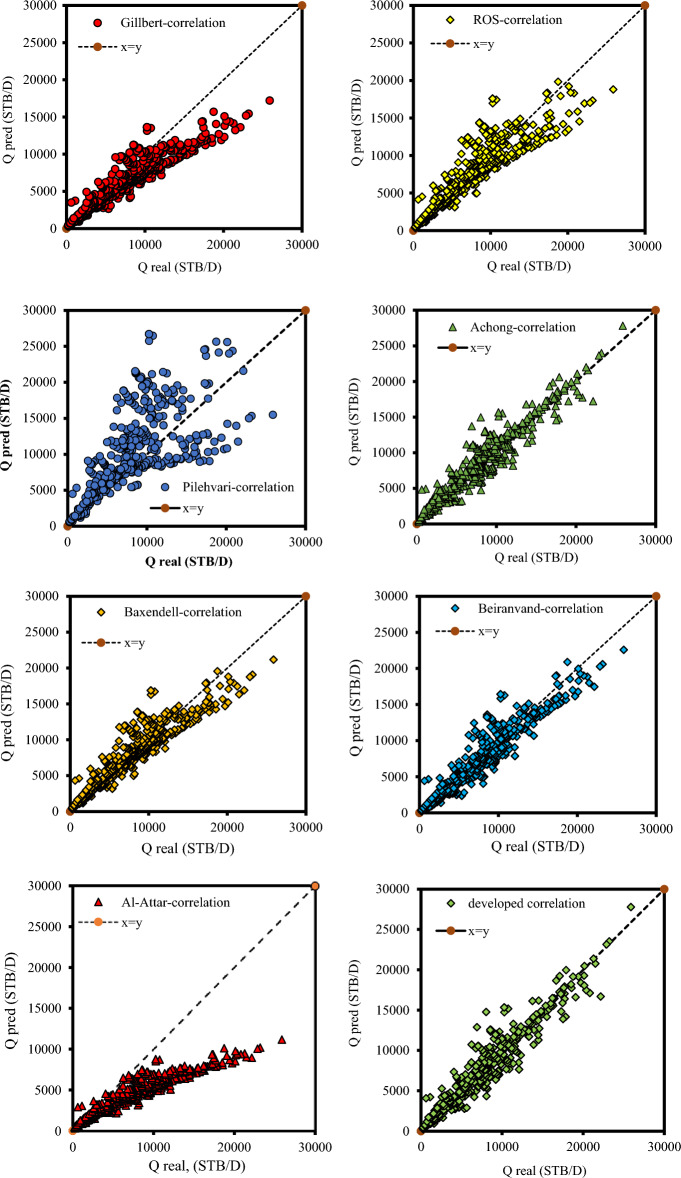
Cross-plot for correlation developed by Gilbert, Ros, Pilehvari, Achong, Baxendell, Beiranvand, Al-Attar and developed correlation in this study.

Furthermore, Fig. 4 is plotted to evaluate the performance of different correlations. As seen in Fig. 4, all correlations proposed for an estimated liquid rate through wellhead chokes showed weak performance. The Gilbert correlation predicts the flow rate is lower than its actual value. Under these conditions, the relative error could be a positive number, and expectations go astray from the proper values. Also, the Pilehvari model overestimates the real data points. In other words, this model tends to predict values to be larger than the real values. In this situation, the relative error could be a negative number, and forecasts veer off from the right values. It is obvious that Ros, Achong, Beiranvand, and Baxendell are models that suffer from a random error in anticipating real value and show poor performance in estimating the liquid rate. It can also be concluded from the Figure that Gilbert and Pilehvari are the models with the least accuracy with the most considerable AAPRE value among all the correlations proposed for estimating the liquid flow through oil field chokes.

**Figure 5:**
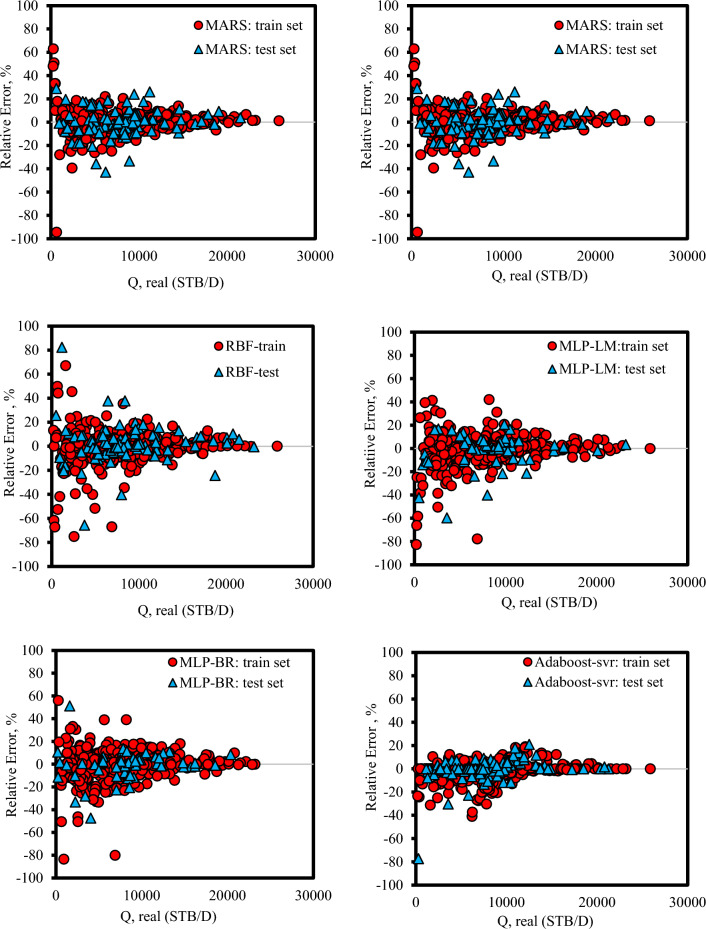
Percent relative error distributions for various intelligence models compared to real flow rate data.

**Figure 6:**
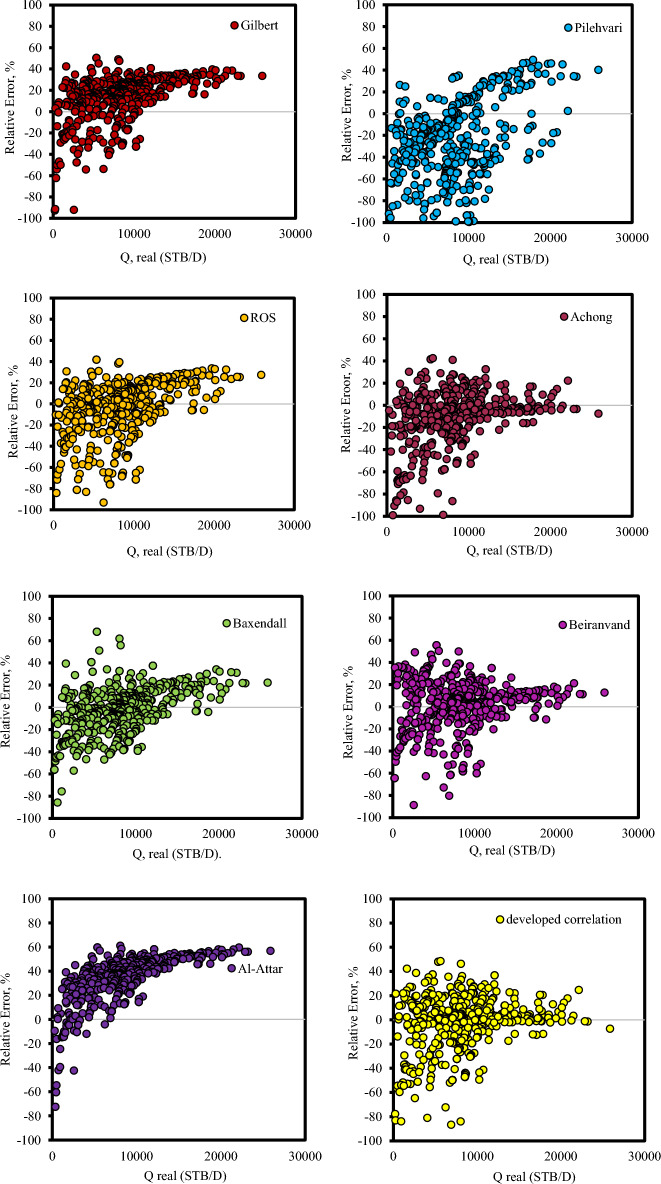
Percent Relative Error different correlation versus real flow rate data.

Figures 5 and 6 illustrate the percent relative error distribution versus the real flow rate for the AI models and correlations to determine the error trend of the predictive models when an independent variable is increased. Concerning Figs. 5 and 6, it can be concluded that AI models have much higher accuracy than the presented correlations.

The data points lie close to the zero-error line regardless of the change in their value. Moreover, these Figures show that by increasing the value of the liquid rate, there is no error trend in this plot, which means that the developed models are suitable for using any range of data. It should be noted that the training phase of these models was developed based on a sufficient amount of data.

Furthermore, the cumulative relative frequency of data (with absolute relative errors below specific increasing values) is plotted against absolute relative error (ARE%) to quantify the number of data that the model can accurately predict. To find cumulative frequencies, it is first necessary to sort the column of the absolute relative errors in ascending order, then the relative frequency of each row is calculated. Relative frequency is obtained by dividing the number of rows by the number of total data. Then, cumulative frequency versus absolute relative error is plotted^54^.

**Figure 7:**
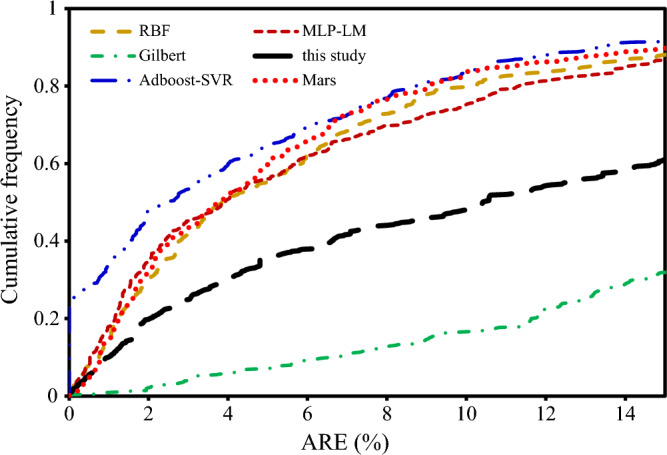
Cumulative frequency error for intelligence models and other correlations.

Figure 7 illustrates the cumulative frequency error versus ARE % for AI models consisting of Adaboost-SVR, MARS, RBF, MLP-LM, and developed correlations consisting of Gilbert and correlation in this study. As seen in Figure, the developed AI models performed better in estimating the liquid flow compared to the others.

correlation studied in this research. The Adaboost-SVR model is the most accurate model among the developed artificial intelligence models showing 91% of the full data set with 15% ARE. It can also be deduced from Fig. 7 that the developed correlations in this study with four coefficients estimate approximately 60% of data with 15% ARE. Regarding correlations, the correlation developed by Gilbert demonstrated poor performance.

**Figure 8:**
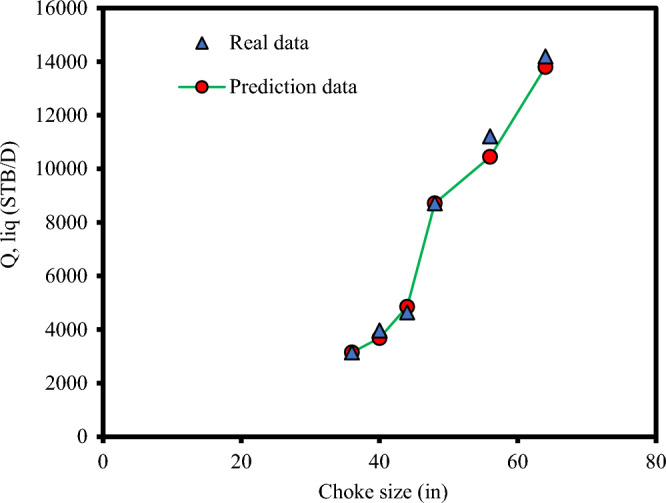
Choke size trend analysis of liquid rate through the choke based on the results of the Adaboost-SVR.

Furthermore, Fig. 8 demonstrates the trend plots of liquid rate in oil field chokes at different choke sizes by the Adaboost-SVR model. As seen in this Figure, there is a very good match between the real and predicted values.

**Figure 9:**
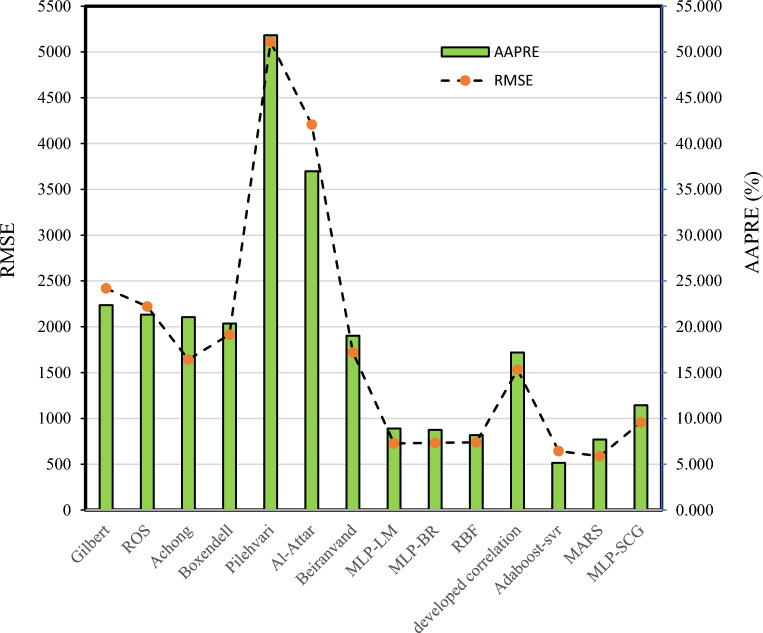
Comparison of AAPRE and RMSE for developed intelligent models and other correlations.

The comparison of AAPRE and RMSE between the proposed AI models and other correlations is shown in Fig. 9. As seen in this Figure, the lowest value of AAPRE and RMSE is related to the Adabost-SVR model.

### Sensitivity analysis

**Figure 10:**
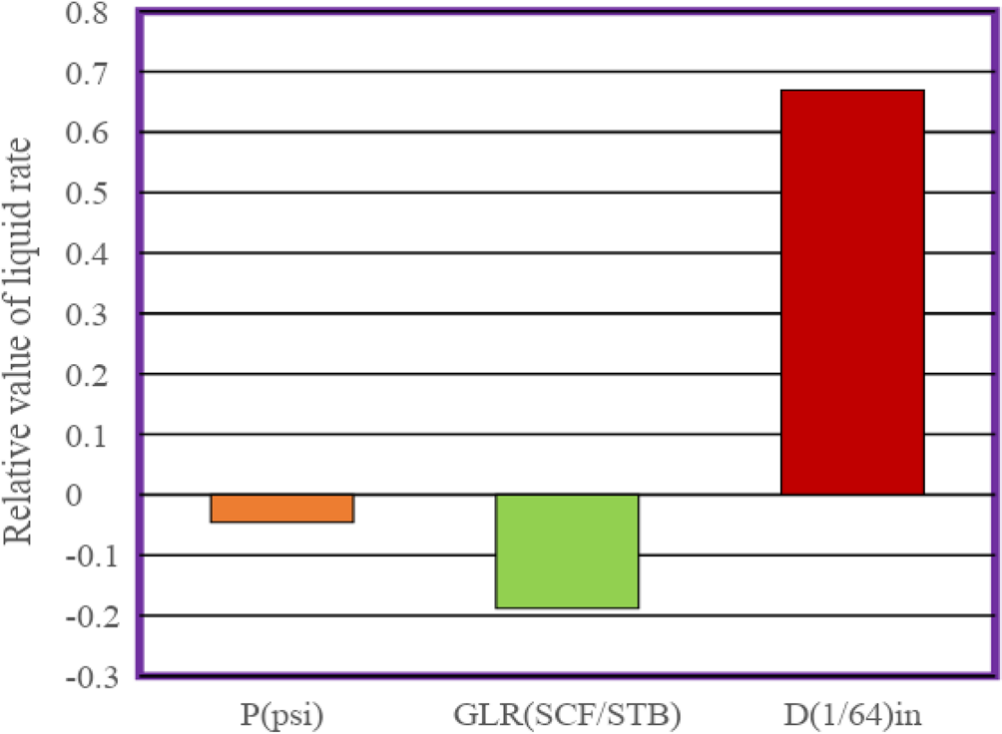
The relative importance of each input on the liquid rate.

Sensitivity analysis of the input parameters was performed in estimating the liquid flow by using Eq. (30). To this end, input data points and real liquid flow rate data were used. This diagram shows the effect of inputs on the liquid flow rate through the choke, which is based on the Pearson relationship. This is defined as follows^26,55^:

30 $$r=\frac{\sum_{i=0}^{n}({I}_{k}-{\underline{I}}_{k})({O}_{i}-\overline{O })}{\sqrt{\sum_{i=0}^{n}{({I}_{k}-{\overline{I} }_{k})}^{2}}\sum_{i=0}^{n}{({O}_{i}-\overline{O })}^{2}}$$

where I_k_ Indicates the input value of the k number of the model (P_wh_, D (1/64), GLR, and Q_L_) and ${\overline{I} }_{k}$ indicates the average value for the input variable k number of the model. O and $\overline{O }$ predicted liquid flow rate and the average predicted liquid flow rate, respectively. also ${I}_{ki}$ shows the amount of k-number input data^25^. Figure 10 illustrates the relative effect of input parameters on the liquid flow rate. This figure demonstrates that the input variable, such as the choke size, exerts a positive influence on the target value. Conversely, the output variable is adversely affected by both P_wh_ and GLR. This implies that any rise in P_wh_ or GLR would lead to a reduction in the liquid flow rate in chokes. As can be seen from this Figure, the largest effect on the liquid flow rate is related to the choke size. Furthermore, the lowest r-value among the input variables considered is − 0.045, which suggests that the gas–liquid ratio has the least impact on the flow rate.

### Outlier diagnostics and model reliability assessment

To find suspicious and out-of-bounds data, a William diagram is drawn using the leverage technique^56^. Such data are not necessarily non-standard data, and their proper P_wh_ range, D_c_, and GLR may differ from other data in a valid range. Data with a hat between 0 and an H* and standardized residual (SR) between −3 and 3 are valid data. Also, data with SR values greater than 3 or lower than −3 are lab-suspicious (regardless of their hat value), and data with Hat higher than Hat* and SR between − 3 and 3 are outside the model scope^57,58^. The SR, Hat*, and H are represented as follows^59^:

31 $$SR=\frac{(outputs-targets)}{({(1-h)}^{0.5}\times RMSE)}$$

32 $${Hat}^{*}=\frac{3(number~ of ~input~ data+1)}{number ~of ~data~ point}$$

33 $$H=X{{(X}^{t}X)}^{-1}{X}^{t}$$

**Figure 11:**
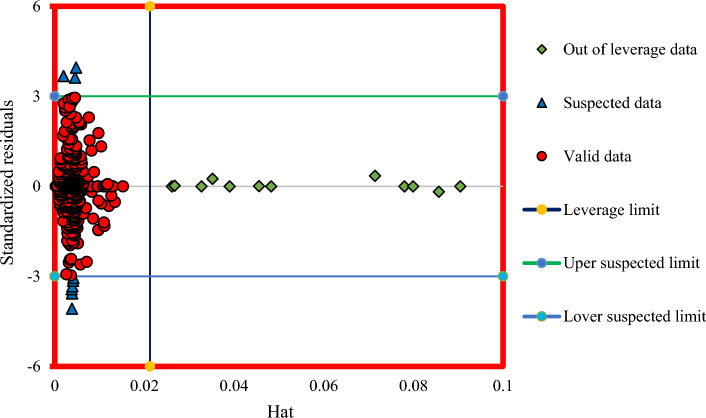
William’s diagram of the proposed Adaboost-SVR model for determining range.

H is defined as a matrix (k × j), in which k and j determine the total number of data and the model parameters, respectively, and t is the concept of transposition. Using the main elements of the matrix diameter, the relationship between each point is obtained and finally, the suspicious data are calculated. Figure 11 illustrates the Williams chart for Adaboost-SVR model^60^. According to the graph, the number of data points out of leverage data is insignificant, affecting the model accuracy considerably, and most of the used data is in the valid zone of the Williams chart. As depicted in Fig. 11 most of the data points are situated within the range of 0 ≤ H ≤ H ∗ and − 3 ≤ R ≤ 3. Data points with lower values of R and H demonstrate higher reliability. Therefore, the identification of data points outside the model's intended scope amounted to a mere 2.1%, which is insignificant when considering the substantial volume of data points used during the model's development. These findings indicate that the proposed Adaboost-SVR model exhibits high reliability.

## Conclusions

In this study, the liquid rate in chokes was modeled using 565 datasets including P_wh_, GLR, and D_c_. Six intelligent models were developed for forecasting the liquid rate. Additionally, developed an empirical equation with four coefficients based on P_wh_, GLR, and D_c_. Statistical analysis confirms that all the developed models in this study can properly estimate the liquid rate through oilfield chokes. Nevertheless, the accuracy of the different models can be ranked as follows:

Adaboost-SVR > MARS > RBF > MLP-LM > MLP-BR > MLP-SCG.

The Adabboost-SVR model is the most precise compared to other intelligent models. The statistical parameters for this model are: R^2^ of 0.9784; RMSE of 643.38; APRE of − 1.5%, and AAPRE of 5.15%. The correlation developed with four coefficients showed the best performance among the earlier correlations in this work (Supplementary file). Furthermore, the results of sensitivity analysis indicated that D_c_ has a positive effect and owns the highest influence on liquid rate through chokes, while GLR and P_wh_ have a negative effect. Finally, outlier detection applying the leverage approach revealed that only 2.1% of the real data points are doubtful.

### Supplementary Information

Supplementary Information.

## Data Availability

The datasets used during the current study are available as a Supplementary file.

## Abbreviations

NN: Neural network
MARS: Multivariate adaptive regression spline
Adaboost-SVR: Adaptive boosting-support vector machine
R^2^: Coefficient of determination
SCG: Scaled conjugate gradient
GLR: Gas to liquid ratio
RMSE: Root mean square error
LM: Levenberg–Marquardt
AI: Artificial intelligence
RBF-GM: Radial basis function-genetic model
MLP: Multilayer perceptron
ANN: Artificial neural network
BR: Bayesian-regularization
APRE%: Average percent relative error
RBF: Radial basis function
P_wh_: Wellhead pressure
SD: Standard deviation
GLR: Gas-to-liquid ratio
SVM: Support vector machine
Dc: Choke size
SR: Standardized residual
γo: Oil specific gravity
AAPRE%: Average absolute percent relative error
γg: Gas specific gravity
T: Temperature
W.C: Water cut
