# Supplementary material for: Modeling liquid rate through wellhead chokes using machine learning techniques
Source: Sci Rep. 2024 Mar 23;14:6945. doi: 10.1038/s41598-024-54010-2 (PMC10960849; doi:10.1038/s41598-024-54010-2)
Supplement: Supplementary file 1 — Supplementary Information. [file 41598_2024_54010_MOESM1_ESM.docx]

**Supplementary data**

Table s1: datasets used for predicting liquid rate through wellhead chokes.

| No. | P(psi) | GLR(SCF/D) | D (1/64) in | Qliq,exp(STB/D) |
| --- | --- | --- | --- | --- |
| 1 | 784 | 69 | 58.88 | 25878 |
| 2 | 1521 | 715 | 72 | 22150 |
| 3 | 490 | 63 | 64 | 21500 |
| 4 | 452 | 66 | 64 | 19710 |
| 5 | 444 | 60 | 64 | 20100 |
| 6 | 740 | 101 | 64 | 23200 |
| 7 | 720 | 99 | 64 | 22870 |
| 8 | 670 | 99 | 64 | 21270 |
| 9 | 442 | 67 | 64 | 18555 |
| 10 | 575 | 91 | 64 | 19399 |
| 11 | 450 | 73 | 64 | 17700 |
| 12 | 1496 | 904 | 80 | 20805 |
| 13 | 549 | 91 | 64 | 18500 |
| 14 | 724 | 119 | 64 | 20150 |
| 15 | 494.5 | 36 | 51.2 | 17888 |
| 16 | 439 | 80 | 64 | 16300 |
| 17 | 1484 | 868 | 72 | 17829 |
| 18 | 1425 | 813 | 80 | 20361 |
| 19 | 1484 | 893 | 72 | 17511 |
| 20 | 439 | 84 | 64 | 15700 |
| 21 | 488 | 93 | 64 | 16200 |
| 22 | 475 | 91 | 64 | 16000 |
| 23 | 519 | 41 | 51.2 | 17100 |
| 24 | 649 | 121 | 64 | 17700 |
| 25 | 423 | 82 | 64 | 15300 |
| 26 | 677 | 131 | 64 | 17600 |
| 27 | 513 | 42 | 51.2 | 16666 |
| 28 | 549 | 111 | 64 | 16000 |
| 29 | 675 | 136 | 64 | 17065 |
| 30 | 616 | 126 | 64 | 16450 |
| 31 | 1573 | 907 | 80 | 20123 |
| 32 | 1510 | 959 | 80 | 19057 |
| 33 | 1430 | 767 | 72 | 17290 |
| 34 | 467 | 98 | 64 | 14870 |
| 35 | 626 | 129 | 64 | 16380 |
| 36 | 2430 | 2018.9 | 54 | 12086.5 |
| 37 | 2430 | 2018.9 | 54 | 12086.5 |
| 38 | 830 | 380 | 80 | 17710 |
| 39 | 2445 | 1049.9 | 52 | 14480.8 |
| 40 | 444 | 97 | 64 | 14300 |
| 41 | 454 | 102 | 64 | 14200 |
| 42 | 1448 | 892 | 64 | 13878 |
| 43 | 693 | 151 | 64 | 16200 |
| 44 | 426 | 102 | 64 | 13400 |
| 45 | 559 | 127 | 64 | 14800 |
| 46 | 461 | 108 | 64 | 13700 |
| 47 | 1550 | 853 | 64 | 14446 |
| 48 | 673 | 157 | 64 | 15370 |
| 49 | 603 | 142 | 64 | 14760 |
| 50 | 419 | 102 | 64 | 13000 |
| 51 | 429 | 108 | 64 | 12900 |
| 52 | 628 | 150 | 64 | 14800 |
| 53 | 2085 | 1833.7 | 60 | 12050 |
| 54 | 1190 | 875 | 60 | 10886 |
| 55 | 913 | 380 | 64 | 13269 |
| 56 | 479 | 125 | 64 | 12900 |
| 57 | 1540 | 660 | 64 | 15508 |
| 58 | 2275 | 1753 | 40 | 8062.5 |
| 59 | 588.7 | 319 | 51.2 | 8200 |
| 60 | 1510 | 1021 | 80 | 17450 |
| 61 | 1132 | 812 | 60 | 10515 |
| 62 | 420 | 118 | 64 | 11800 |
| 63 | 479 | 131 | 64 | 12400 |
| 64 | 523 | 144 | 64 | 12720 |
| 65 | 430 | 119 | 64 | 11900 |
| 66 | 708 | 188 | 64 | 14200 |
| 67 | 1479 | 895 | 56 | 10966 |
| 68 | 2185 | 1833.7 | 58 | 11367.3 |
| 69 | 1808 | 1805 | 68 | 12311.7 |
| 70 | 460 | 133 | 64 | 11800 |
| 71 | 1176 | 944 | 80 | 14577 |
| 72 | 2495 | 1050.9 | 40 | 9442.6 |
| 73 | 748 | 122 | 56.32 | 14500 |
| 74 | 1380 | 783.5 | 68 | 13911 |
| 75 | 1001 | 380 | 56 | 11218 |
| 76 | 655 | 76 | 51.2 | 13740 |
| 77 | 517 | 173 | 64 | 11300 |
| 78 | 2208 | 1805 | 50 | 9218.3 |
| 79 | 2208 | 1805 | 50 | 9218.3 |
| 80 | 1566 | 708.5 | 64 | 14474 |
| 81 | 1143 | 777 | 64 | 11008 |
| 82 | 2285 | 1695.2 | 50 | 9584.7 |
| 83 | 464 | 149 | 64 | 11000 |
| 84 | 1411 | 803 | 56 | 10495 |
| 85 | 2158 | 1701.6 | 52 | 9624.2 |
| 86 | 2158 | 1701.6 | 52 | 9624.2 |
| 87 | 528 | 172 | 64 | 11300 |
| 88 | 2775 | 1753 | 40 | 8062.5 |
| 89 | 2775 | 1753 | 40 | 8062.5 |
| 90 | 1499 | 773 | 80 | 18740 |
| 91 | 2310 | 1696.9 | 50 | 9424.2 |
| 92 | 1523 | 930 | 80 | 17356 |
| 93 | 1215 | 1117 | 80 | 13315 |
| 94 | 1523 | 939 | 80 | 17230 |
| 95 | 1858 | 1785 | 68 | 11925 |
| 96 | 1858 | 1785 | 68 | 11925 |
| 97 | 511 | 178 | 64 | 10670 |
| 98 | 549 | 188 | 64 | 11000 |
| 99 | 2023 | 1709.7 | 64 | 11809.4 |
| 100 | 2023 | 1709.7 | 64 | 11809.4 |
| 101 | 535.1 | 189 | 64 | 10720 |
| 102 | 805 | 340 | 64 | 11447 |
| 103 | 2285 | 1939.2 | 58 | 10652.6 |
| 104 | 508 | 177 | 64 | 10500 |
| 105 | 2200 | 1518.2 | 31 | 5353.2 |
| 106 | 2200 | 1518.2 | 31 | 5353.2 |
| 107 | 2200 | 1040 | 47 | 9860 |
| 108 | 2200 | 1040 | 47 | 9860 |
| 109 | 2800 | 1753 | 40 | 7740 |
| 110 | 2800 | 1753 | 40 | 7740 |
| 111 | 551 | 249 | 64 | 9700 |
| 112 | 2920 | 1985.4 | 48 | 9621.6 |
| 113 | 501 | 189 | 64 | 10049 |
| 114 | 2920 | 2010.9 | 48 | 9499.6 |
| 115 | 2920 | 2010.9 | 48 | 9499.6 |
| 116 | 2920 | 2010.9 | 48 | 9499.6 |
| 117 | 2208 | 1699.9 | 52 | 9246 |
| 118 | 2208 | 1699.9 | 52 | 9246 |
| 119 | 1269 | 804 | 72 | 13233 |
| 120 | 1300 | 851 | 72 | 13151 |
| 121 | 3575 | 4699.6 | 36 | 5632.8 |
| 122 | 480 | 192 | 64 | 9600 |
| 123 | 2285 | 1839.2 | 58 | 10652.6 |
| 124 | 518 | 194 | 64 | 10100 |
| 125 | 2285 | 1841 | 58 | 10642 |
| 126 | 2280 | 1818.2 | 40 | 6566.6 |
| 127 | 537 | 242 | 64 | 9458 |
| 128 | 506 | 197 | 64 | 9834 |
| 129 | 2285 | 1798.4 | 46 | 7797.8 |
| 130 | 512 | 201 | 64 | 9800 |
| 131 | 558 | 208 | 64 | 10300 |
| 132 | 513 | 206 | 64 | 9700 |
| 133 | 621 | 92 | 51.2 | 11390 |
| 134 | 1485 | 950 | 64 | 11552 |
| 135 | 1190 | 1501 | 64 | 8124 |
| 136 | 1200 | 1013 | 80 | 13266 |
| 137 | 2350 | 1040 | 47 | 10075 |
| 138 | 541 | 214 | 64 | 9900 |
| 139 | 509 | 201 | 64 | 9700 |
| 140 | 494 | 238 | 64 | 8860 |
| 141 | 1900 | 1805 | 68 | 11600.2 |
| 142 | 689 | 261 | 64 | 10940 |
| 143 | 2179 | 1646.7 | 50 | 8580 |
| 144 | 2695 | 1048.9 | 36 | 7584.9 |
| 145 | 384 | 68 | 51.2 | 8830 |
| 146 | 502 | 210 | 64 | 9320 |
| 147 | 2385 | 1693.5 | 50 | 8981.2 |
| 148 | 722.1 | 275 | 64 | 11000 |
| 149 | 378 | 66 | 51.2 | 8800 |
| 150 | 505 | 211 | 64 | 9300 |
| 151 | 1399 | 1118 | 48 | 6817 |
| 152 | 2308 | 1801.4 | 50 | 8461.8 |
| 153 | 2308 | 1801.4 | 50 | 8461.8 |
| 154 | 511 | 229 | 64 | 9000 |
| 155 | 2800 | 2025 | 54 | 10413.7 |
| 156 | 499 | 222 | 64 | 8905 |
| 157 | 378 | 69 | 51.2 | 8500 |
| 158 | 1368 | 829 | 72 | 13425 |
| 159 | 1425 | 888.5 | 68 | 12297 |
| 160 | 2400 | 1040 | 47 | 9920 |
| 161 | 2073 | 1665 | 64 | 11457.3 |
| 162 | 2073 | 1665 | 64 | 11457.3 |
| 163 | 2300 | 1040 | 47 | 9570 |
| 164 | 2300 | 1040 | 47 | 9570 |
| 165 | 493 | 230 | 64 | 8630 |
| 166 | 1500 | 1093 | 48 | 7016 |
| 167 | 372 | 74 | 51.2 | 8020 |
| 168 | 1300 | 782 | 64 | 10846 |
| 169 | 1451 | 842 | 72 | 13832 |
| 170 | 2850 | 1753 | 40 | 7095 |
| 171 | 473 | 239 | 64 | 8100 |
| 172 | 2071 | 1423.9 | 48 | 7836.9 |
| 173 | 2300 | 1518.2 | 31 | 4684 |
| 174 | 2300 | 1518.2 | 31 | 4684 |
| 175 | 2076 | 1423.9 | 48 | 7811.3 |
| 176 | 573 | 279 | 64 | 8700 |
| 177 | 1417 | 863 | 72 | 13270 |
| 178 | 543 | 280 | 64 | 8300 |
| 179 | 582 | 284 | 64 | 8700 |
| 180 | 1326 | 813 | 44 | 6157 |
| 181 | 2745 | 1048.9 | 36 | 7165.8 |
| 182 | 2745 | 1048.9 | 36 | 7165.8 |
| 183 | 2745 | 1048.9 | 36 | 7165.8 |
| 184 | 2745 | 1048.9 | 36 | 7165.8 |
| 185 | 2745 | 1048.9 | 36 | 7165.8 |
| 186 | 482 | 244 | 64 | 8000 |
| 187 | 2300 | 1271.2 | 31 | 4844.5 |
| 188 | 580 | 297 | 64 | 8500 |
| 189 | 525 | 269 | 64 | 8200 |
| 190 | 2300 | 1272.5 | 31 | 4839.7 |
| 191 | 1435 | 966 | 72 | 12673 |
| 192 | 2350 | 1040 | 47 | 9425 |
| 193 | 2385 | 1800.2 | 46 | 7292.3 |
| 194 | 2385 | 1800.2 | 46 | 7292.3 |
| 195 | 2945 | 2010.9 | 48 | 8715.8 |
| 196 | 371 | 205 | 64 | 7000 |
| 197 | 664 | 336 | 64 | 8900 |
| 198 | 573 | 300 | 64 | 8300 |
| 199 | 561 | 296 | 64 | 8200 |
| 200 | 2358 | 1805 | 50 | 8058.3 |
| 201 | 2358 | 1805 | 50 | 8058.3 |
| 202 | 1958 | 1805 | 68 | 11151.7 |
| 203 | 549 | 299 | 64 | 7992 |
| 204 | 1743 | 1785 | 68 | 10157.4 |
| 205 | 1291 | 911 | 72 | 11780 |
| 206 | 1432 | 876 | 60 | 9784 |
| 207 | 325 | 200 | 64 | 6300 |
| 208 | 600 | 125 | 51.2 | 8910 |
| 209 | 1180 | 915 | 64 | 8987 |
| 210 | 1830 | 1916.4 | 68 | 10180.3 |
| 211 | 593.1 | 327 | 64 | 8100 |
| 212 | 2645 | 1051.9 | 52 | 11949.4 |
| 213 | 1716 | 660 | 48 | 9041 |
| 214 | 2645 | 1052.5 | 52 | 11943.5 |
| 215 | 1203 | 950 | 48 | 5852 |
| 216 | 1404 | 921 | 44 | 5871 |
| 217 | 2700 | 1814.1 | 62 | 12502.6 |
| 218 | 970 | 774 | 48 | 5400 |
| 219 | 2385 | 1800.2 | 47 | 7292.3 |
| 220 | 2385 | 1800.2 | 47 | 7292.3 |
| 221 | 2385 | 1800.2 | 47 | 7292.3 |
| 222 | 2385 | 1800.2 | 47 | 7292.3 |
| 223 | 600 | 333 | 64 | 8045 |
| 224 | 1758 | 1785 | 68 | 10065.5 |
| 225 | 475 | 267 | 64 | 7300 |
| 226 | 554 | 332 | 64 | 7500 |
| 227 | 2500 | 1040 | 47 | 9610 |
| 228 | 593 | 331 | 64 | 7934 |
| 229 | 2400 | 1040 | 47 | 9280 |
| 230 | 2400 | 1040 | 47 | 9280 |
| 231 | 2400 | 1040 | 47 | 9280 |
| 232 | 2400 | 1040 | 47 | 9280 |
| 233 | 2400 | 1040 | 47 | 9280 |
| 234 | 2400 | 1040 | 47 | 9280 |
| 235 | 2400 | 1040 | 47 | 9280 |
| 236 | 2400 | 1040 | 47 | 9280 |
| 237 | 2400 | 1040 | 47 | 9280 |
| 238 | 2400 | 1040 | 47 | 9280 |
| 239 | 2185 | 1791.3 | 54 | 8329.2 |
| 240 | 1397 | 884 | 68 | 11437 |
| 241 | 899 | 818 | 64 | 7424 |
| 242 | 2136 | 1425.3 | 48 | 7496.3 |
| 243 | 2279 | 1646.7 | 50 | 7888.6 |
| 244 | 2279 | 1646.7 | 50 | 7888.6 |
| 245 | 2279 | 1646.7 | 50 | 7888.6 |
| 246 | 2279 | 1646.7 | 50 | 7888.6 |
| 247 | 2279 | 1646.7 | 50 | 7888.6 |
| 248 | 560 | 347 | 64 | 7345 |
| 249 | 580 | 352 | 64 | 7500 |
| 250 | 571 | 343 | 64 | 7489 |
| 251 | 2350 | 1271.2 | 31 | 4498.5 |
| 252 | 568 | 348 | 64 | 7400 |
| 253 | 602 | 325 | 64 | 8002 |
| 254 | 1830 | 1816.4 | 68 | 10180.3 |
| 255 | 1830 | 1816.4 | 68 | 10180.3 |
| 256 | 1390 | 804.6 | 68 | 11812 |
| 257 | 616 | 335 | 64 | 8000 |
| 258 | 626 | 220 | 56.32 | 8000 |
| 259 | 910 | 738 | 48 | 5012 |
| 260 | 1130 | 835 | 72 | 10580 |
| 261 | 1650 | 916.5 | 40 | 5503 |
| 262 | 3825 | 5706.6 | 36 | 4201.5 |
| 263 | 2300 | 1230.4 | 31 | 4337.9 |
| 264 | 1058 | 470 | 48 | 6756 |
| 265 | 2760 | 1753 | 48 | 8256 |
| 266 | 2300 | 1250 | 31 | 4269.8 |
| 267 | 920 | 679 | 40 | 3972 |
| 268 | 1421 | 814 | 44 | 5811 |
| 269 | 505 | 358 | 64 | 6400 |
| 270 | 540 | 356 | 64 | 6780 |
| 271 | 508 | 361 | 64 | 6401 |
| 272 | 496 | 355 | 64 | 6320 |
| 273 | 2335 | 1839.2 | 58 | 9402.4 |
| 274 | 538 | 400 | 64 | 6400 |
| 275 | 1450 | 759 | 32 | 3812 |
| 276 | 1190 | 937 | 80 | 12308 |
| 277 | 467 | 348 | 64 | 6050 |
| 278 | 511 | 368 | 64 | 6350 |
| 279 | 3840 | 5599 | 36 | 4090.4 |
| 280 | 1211 | 340 | 48 | 8630 |
| 281 | 1455 | 839 | 64 | 10621 |
| 282 | 2180 | 1421.1 | 48 | 7292.7 |
| 283 | 2180 | 1421.1 | 48 | 7292.7 |
| 284 | 1320 | 877 | 40 | 4534 |
| 285 | 1421 | 885 | 60 | 9075 |
| 286 | 2181 | 1425.3 | 48 | 7265.8 |
| 287 | 920 | 619 | 40 | 3972 |
| 288 | 1761 | 660 | 40 | 6450 |
| 289 | 510 | 380 | 64 | 6180 |
| 290 | 1435 | 827 | 56 | 8387 |
| 291 | 2970 | 1964.3 | 48 | 8120.1 |
| 292 | 2970 | 1964.3 | 48 | 8120.1 |
| 293 | 2545 | 884.2 | 48 | 10395.7 |
| 294 | 2545 | 884.2 | 48 | 10395.7 |
| 295 | 2725 | 1814.1 | 62 | 12021.7 |
| 296 | 2970 | 1985.4 | 48 | 8033.9 |
| 297 | 2970 | 1985.4 | 48 | 8033.9 |
| 298 | 2970 | 1985.4 | 48 | 8033.9 |
| 299 | 2970 | 1985.4 | 48 | 8033.9 |
| 300 | 2970 | 1985.4 | 48 | 8033.9 |
| 301 | 2970 | 1985.4 | 48 | 8033.9 |
| 302 | 2970 | 1985.4 | 48 | 8033.9 |
| 303 | 2970 | 1985.4 | 48 | 8033.9 |
| 304 | 2970 | 1985.4 | 48 | 8033.9 |
| 305 | 2191 | 1425.3 | 48 | 7214.6 |
| 306 | 2400 | 1272.5 | 31 | 4148.3 |
| 307 | 2400 | 1272.5 | 31 | 4148.3 |
| 308 | 2192 | 1425.3 | 48 | 7209.5 |
| 309 | 1019 | 380 | 32 | 3733 |
| 310 | 4045 | 4015 | 24 | 2711.4 |
| 311 | 2730 | 2023 | 60 | 10754.3 |
| 312 | 2545 | 890.3 | 48 | 10323.6 |
| 313 | 569 | 391 | 64 | 6600 |
| 314 | 1334 | 753 | 44 | 5504.5 |
| 315 | 3668 | 5706.6 | 36 | 3761.3 |
| 316 | 466 | 388 | 64 | 5578 |
| 317 | 2970 | 2006.9 | 48 | 7947.7 |
| 318 | 1193 | 975 | 48 | 5101 |
| 319 | 2198 | 1425.3 | 48 | 7178.8 |
| 320 | 750 | 701 | 56 | 5125 |
| 321 | 2400 | 1279.2 | 31 | 4074.2 |
| 322 | 2279 | 1646.7 | 52 | 7888.6 |
| 323 | 1453 | 737 | 32 | 3647 |
| 324 | 900 | 811 | 72 | 8400 |
| 325 | 457 | 393 | 64 | 5417 |
| 326 | 2280 | 1816.4 | 48 | 6573.1 |
| 327 | 2279 | 1646.7 | 50 | 7362.7 |
| 328 | 2690 | 1052.5 | 52 | 11380.7 |
| 329 | 2206 | 1425.3 | 48 | 7137.8 |
| 330 | 2207 | 1422.5 | 48 | 7146.9 |
| 331 | 2350 | 1236.5 | 31 | 4008.3 |
| 332 | 2208 | 1425.3 | 48 | 7127.6 |
| 333 | 2209 | 1422.5 | 48 | 7136.7 |
| 334 | 2280 | 3660 | 48 | 4700 |
| 335 | 2385 | 1772.1 | 50 | 7321.4 |
| 336 | 1344 | 691 | 44 | 5617 |
| 337 | 1379 | 698 | 56 | 8545 |
| 338 | 2216 | 1425.3 | 48 | 7086.6 |
| 339 | 455 | 364 | 61.44 | 5118 |
| 340 | 2217 | 1425.3 | 48 | 7081.5 |
| 341 | 421 | 390 | 61.44 | 4632 |
| 342 | 436 | 408 | 61.44 | 4668 |
| 343 | 456 | 381 | 61.44 | 5002 |
| 344 | 1250 | 918 | 72 | 10494 |
| 345 | 1795 | 660 | 32 | 4331 |
| 346 | 1880 | 1816.4 | 68 | 9779.5 |
| 347 | 446 | 372 | 61.44 | 4920 |
| 348 | 1880 | 1820 | 68 | 9760 |
| 349 | 1880 | 1820 | 68 | 9760 |
| 350 | 50 | 270 | 80 | 1615 |
| 351 | 990 | 702 | 32 | 2618 |
| 352 | 456 | 386 | 61.44 | 4917 |
| 353 | 775 | 225 | 51.2 | 7530 |
| 354 | 810 | 732 | 56 | 5125 |
| 355 | 1660 | 900 | 32 | 3500 |
| 356 | 1230 | 816 | 48 | 5430 |
| 357 | 722 | 348 | 56.32 | 6667 |
| 358 | 1486 | 340 | 40 | 7207 |
| 359 | 727 | 347 | 56.32 | 6700 |
| 360 | 54 | 107 | 80 | 2286 |
| 361 | 441 | 385 | 61.44 | 4724 |
| 362 | 435 | 405 | 61.44 | 4554 |
| 363 | 421 | 387 | 61.44 | 4520 |
| 364 | 1020 | 819 | 48 | 4553 |
| 365 | 1150 | 957 | 40 | 3500 |
| 366 | 1299 | 1568 | 32 | 2217 |
| 367 | 2480 | 1816.4 | 40 | 4969.9 |
| 368 | 1653 | 340 | 32 | 5371 |
| 369 | 2480 | 1818.2 | 40 | 4965 |
| 370 | 2480 | 1818.2 | 40 | 4965 |
| 371 | 2480 | 1818.2 | 40 | 4955 |
| 372 | 420 | 410 | 61.44 | 4330 |
| 373 | 310 | 440 | 56 | 2804 |
| 374 | 583 | 272 | 30.72 | 2320 |
| 375 | 1240 | 920 | 36 | 3152 |
| 376 | 411 | 414 | 61.44 | 4210 |
| 377 | 417 | 415 | 61.44 | 4250 |
| 378 | 265 | 479 | 64 | 2885 |
| 379 | 950 | 1600 | 36 | 1961 |
| 380 | 261 | 355 | 40 | 1600 |
| 381 | 1000 | 712 | 40 | 3400 |
| 382 | 2400 | 1272.5 | 31 | 3629.2 |
| 383 | 1500 | 900 | 56 | 7800 |
| 384 | 1600 | 900 | 48 | 6300 |
| 385 | 2940 | 3660 | 32 | 2750 |
| 386 | 1050 | 886 | 32 | 2225 |
| 387 | 1650 | 900 | 40 | 4700 |
| 388 | 1330 | 753 | 32 | 2907 |
| 389 | 930 | 639 | 24 | 1513 |
| 390 | 921 | 971 | 40 | 2697 |
| 391 | 980 | 753 | 36 | 2700 |
| 392 | 2329 | 1646.7 | 52 | 7543 |
| 393 | 2329 | 1646.7 | 52 | 7543 |
| 394 | 2329 | 1646.7 | 52 | 7543 |
| 395 | 1387 | 846 | 24 | 1813 |
| 396 | 413 | 410 | 61.44 | 4100 |
| 397 | 1421 | 854 | 60 | 8566 |
| 398 | 1646 | 901 | 44 | 5485.6 |
| 399 | 1252 | 470 | 32 | 3395 |
| 400 | 1200 | 1152 | 32 | 2137 |
| 401 | 2330 | 1816.4 | 48 | 6172.3 |
| 402 | 1400 | 1179 | 32 | 2400 |
| 403 | 419 | 526 | 61.44 | 3610 |
| 404 | 905 | 699 | 24 | 1324 |
| 405 | 2129 | 1616.8 | 60 | 8925.7 |
| 406 | 415 | 560 | 61.44 | 3412 |
| 407 | 912 | 906 | 24 | 1166 |
| 408 | 2780 | 2023 | 60 | 10227.1 |
| 409 | 2825 | 1753 | 48 | 7417.5 |
| 410 | 2825 | 1753 | 48 | 7417.5 |
| 411 | 546 | 930 | 64 | 3600 |
| 412 | 443 | 652 | 61.44 | 3286 |
| 413 | 818 | 507 | 32 | 2100 |
| 414 | 2855 | 2025 | 58 | 9810.8 |
| 415 | 2855 | 2025 | 58 | 9810.8 |
| 416 | 2855 | 2025 | 58 | 9810.8 |
| 417 | 2855 | 2025 | 58 | 9810.8 |
| 418 | 1290 | 2167 | 40 | 2256 |
| 419 | 423 | 656 | 61.44 | 3120 |
| 420 | 433 | 661 | 61.44 | 3160 |
| 421 | 1598 | 500 | 20 | 1700 |
| 422 | 832 | 729 | 40 | 2570 |
| 423 | 970 | 697 | 40 | 3026 |
| 424 | 1350 | 900 | 36 | 3000 |
| 425 | 824 | 243 | 40 | 4500 |
| 426 | 798 | 364 | 51.2 | 5537 |
| 427 | 2975 | 1753 | 40 | 5482.5 |
| 428 | 818 | 467 | 32 | 2100 |
| 429 | 370 | 367 | 40 | 1674 |
| 430 | 3910 | 5599 | 36 | 3166.8 |
| 431 | 2183 | 1098.3 | 48 | 7277.3 |
| 432 | 880 | 891 | 32 | 1600 |
| 433 | 783 | 369 | 51.2 | 5355 |
| 434 | 2935 | 1747 | 32 | 3557 |
| 435 | 133 | 646 | 61.44 | 1004 |
| 436 | 2185 | 3421 | 40 | 2804 |
| 437 | 1837 | 1503 | 16 | 700 |
| 438 | 534 | 409 | 20 | 615 |
| 439 | 1302 | 3792 | 16 | 282 |
| 440 | 2780 | 2025 | 62 | 10632.9 |
| 441 | 424 | 406 | 51.2 | 2715 |
| 442 | 1970 | 1636 | 16 | 649 |
| 443 | 2830 | 2030 | 60 | 10084.8 |
| 444 | 1280 | 960 | 16 | 500 |
| 445 | 2650 | 1040 | 47 | 8555 |
| 446 | 880 | 712 | 48 | 3570 |
| 447 | 650 | 600 | 40 | 1951 |
| 448 | 2380 | 1816.4 | 48 | 5771.5 |
| 449 | 2216 | 1098.3 | 48 | 7107.8 |
| 450 | 833.8 | 477 | 51.2 | 4700 |
| 451 | 350 | 885 | 25.6 | 205 |
| 452 | 342 | 67 | 30.72 | 2039 |
| 453 | 2925 | 1753 | 44 | 6127.5 |
| 454 | 2925 | 1753 | 44 | 6127.5 |
| 455 | 2925 | 1753 | 44 | 6127.5 |
| 456 | 2217 | 1101.6 | 48 | 7081.5 |
| 457 | 373 | 456 | 80 | 5000 |
| 458 | 332 | 742 | 30.72 | 359 |
| 459 | 330 | 84 | 30.72 | 1675 |
| 460 | 395 | 761 | 25.6 | 253 |
| 461 | 294 | 123 | 30.72 | 1136 |
| 462 | 312 | 489 | 30.72 | 446 |
| 463 | 324 | 463 | 30.72 | 490 |
| 464 | 832 | 362 | 46.08 | 4392 |
| 465 | 2224 | 1101.6 | 48 | 7045.7 |
| 466 | 1348 | 824 | 40 | 3412 |
| 467 | 842 | 382 | 46.08 | 4300 |
| 468 | 311 | 176 | 30.72 | 927 |
| 469 | 392 | 316 | 38.4 | 1390 |
| 470 | 893 | 452 | 40 | 3100 |
| 471 | 342 | 133 | 30.72 | 1248 |
| 472 | 392 | 351 | 38.4 | 1289 |
| 473 | 2725 | 1819.2 | 60 | 10072.6 |
| 474 | 453 | 298 | 38.4 | 1678 |
| 475 | 455 | 670 | 43.52 | 1310 |
| 476 | 439 | 400 | 30.72 | 735 |
| 477 | 469.8 | 400 | 38.4 | 1415 |
| 478 | 2825 | 1819.2 | 54 | 8461 |
| 479 | 2825 | 1819.2 | 54 | 8461 |
| 480 | 502 | 292 | 38.4 | 1875 |
| 481 | 462 | 400 | 38.4 | 1370 |
| 482 | 580 | 300 | 38.4 | 2175 |
| 483 | 2323 | 1713.1 | 64 | 9954.1 |
| 484 | 507 | 390 | 38.4 | 1545 |
| 485 | 3850 | 4766.6 | 36 | 2904.3 |
| 486 | 602 | 269 | 38.4 | 2399 |
| 487 | 619 | 363 | 38.4 | 1999 |
| 488 | 529 | 189 | 25.6 | 925 |
| 489 | 650 | 299 | 38.4 | 2390 |
| 490 | 2345 | 915.3 | 48 | 8003.9 |
| 491 | 2875 | 1753 | 48 | 6772.5 |
| 492 | 2050 | 1822.4 | 64 | 8139.2 |
| 493 | 2825 | 1811.9 | 58 | 9412.8 |
| 494 | 2645 | 1050.9 | 52 | 9664.8 |
| 495 | 2825 | 1815.6 | 58 | 9394.1 |
| 496 | 2825 | 1817.4 | 58 | 9384.7 |
| 497 | 3885 | 4444.9 | 36 | 2744.7 |
| 498 | 1677 | 828.1 | 44 | 4645.1 |
| 499 | 881 | 362 | 38.4 | 2570 |
| 500 | 2975 | 1753 | 44 | 5482.5 |
| 501 | 1703 | 828.1 | 44 | 4582.3 |
| 502 | 2825 | 1819.2 | 56 | 8461 |
| 503 | 2825 | 1819.2 | 56 | 8461 |
| 504 | 2825 | 1819.2 | 56 | 8461 |
| 505 | 3030 | 2018.9 | 54 | 7916.1 |
| 506 | 3877 | 2615.6 | 36 | 3596.7 |
| 507 | 3030 | 2020.9 | 54 | 7908.3 |
| 508 | 3030 | 2023 | 54 | 7900.4 |
| 509 | 3030 | 2023 | 54 | 7900.4 |
| 510 | 3835 | 2777.6 | 36 | 3262.6 |
| 511 | 2100 | 1824.3 | 64 | 7860.1 |
| 512 | 1740 | 828.1 | 44 | 4493 |
| 513 | 2075 | 1822.4 | 64 | 7723.4 |
| 514 | 3677 | 2722.1 | 36 | 3065.3 |
| 515 | 3677 | 2722.1 | 36 | 3065.3 |
| 516 | 2480 | 1816.4 | 48 | 4969.9 |
| 517 | 1756 | 833 | 44 | 4427.9 |
| 518 | 3735 | 2777.6 | 36 | 2912.3 |
| 519 | 3735 | 2777.6 | 36 | 2912.3 |
| 520 | 2900 | 2018.9 | 60 | 8979.8 |
| 521 | 1790 | 833 | 44 | 4346.3 |
| 522 | 2423 | 1709.7 | 64 | 9294.6 |
| 523 | 2950 | 1853 | 48 | 5805 |
| 524 | 2423 | 1709.7 | 64 | 9249.6 |
| 525 | 2423 | 1709.7 | 64 | 9249.6 |
| 526 | 2423 | 1709.7 | 64 | 9249.6 |
| 527 | 2423 | 1709.7 | 64 | 9249.5 |
| 528 | 2423 | 1711.4 | 64 | 9240.4 |
| 529 | 2423 | 1713.1 | 64 | 9231.2 |
| 530 | 2433 | 1709.7 | 64 | 9249.6 |
| 531 | 2094 | 1616.8 | 68 | 9167.7 |
| 532 | 2150 | 1824.3 | 64 | 7589 |
| 533 | 2150 | 1824.3 | 64 | 7589 |
| 534 | 2150 | 1824.3 | 64 | 7589 |
| 535 | 2150 | 1824.3 | 64 | 7589 |
| 536 | 2150 | 1826.1 | 64 | 7581.5 |
| 537 | 2677 | 2229.6 | 56 | 6207.5 |
| 538 | 2200 | 1824.3 | 64 | 7318 |
| 539 | 1662 | 186 | 40 | 8030 |
| 540 | 1921 | 828.1 | 44 | 4055.7 |
| 541 | 2780 | 1818.2 | 40 | 2562.6 |
| 542 | 2577 | 1613.9 | 56 | 6753.6 |
| 543 | 2179 | 1646.7 | 68 | 8580 |
| 544 | 2179 | 1646.7 | 68 | 8580 |
| 545 | 2473 | 1661.7 | 64 | 8657.7 |
| 546 | 2250 | 1818.8 | 64 | 7068 |
| 547 | 2250 | 1824.3 | 64 | 7047 |
| 548 | 2250 | 1824.3 | 64 | 7047 |
| 549 | 2250 | 1824.3 | 64 | 7047 |
| 550 | 2250 | 1824.3 | 64 | 7047 |
| 551 | 2250 | 1824.3 | 64 | 7047 |
| 552 | 2250 | 1827.9 | 64 | 7032.9 |
| 553 | 420 | 480 | 64 | 1120 |
| 554 | 2179 | 1616.8 | 68 | 8580 |
| 555 | 2179 | 1616.8 | 68 | 8580 |
| 556 | 2179 | 1616.8 | 68 | 8580 |
| 557 | 2488 | 1661.7 | 64 | 8551.9 |
| 558 | 2523 | 1709.7 | 64 | 8525.2 |
| 559 | 2800 | 1820.4 | 68 | 10746.4 |
| 560 | 763 | 250 | 64 | 6900 |
| 561 | 2725 | 1820.4 | 68 | 10270.5 |
| 562 | 309 | 310 | 64 | 618 |
| 563 | 2677 | 1605.9 | 56 | 6232.2 |
| 564 | 2825 | 1820.4 | 68 | 10258 |
| 565 | 2825 | 1820.4 | 68 | 10258 |

**Description of data scattering by box plot**

Box diagrams can also be used for data scattering and distribution. This chart consists of the minimum, the first quarter, the middle, the third quarter, and the maximum data. Statistical analysis of this chart is given in Table s2.

Table s2. Statistical analysis input variables for Box chart.

| Parameters | P_wh_ (psi) | GLR (STB/D) | D (1/64) in. |
| --- | --- | --- | --- |
| Min | 50 | 36 | 16 |
| Q1 | 593 | 364 | 44 |
| Median | 1500 | 915 | 54 |
| Q2 | 2358 | 1753 | 64 |
| Max | 4045 | 5706.6 | 80 |

The input data box diagram is presented in Figure s1. If the middle line is sloping down the box indicates that the data points are scattered at the beginning of the scale, and if the midline is sloping up the box indicates that the data is scattered at the final of the scale.

| 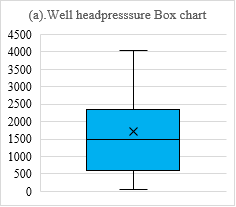 | 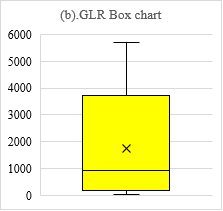 | 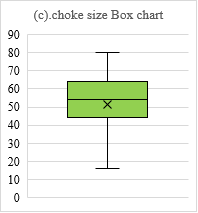 |
| --- | --- | --- |

Figure s1. Box chart plot for input variables. (a). P_wh_, (b). GLR, (C). Dc (1/64).

**Explanation of the models used in this work**

1. **Adaboost_SVR:** "Readers can employ the provided hyperparameters as a starting point for implementing Adaboost_SVR. Detailed information on each parameter is available in Table s3. Utilizing the dataset provided in the Supplementary file, users can model their algorithms, adjusting parameters to suit their specific requirements."

2. **MARS (Multivariate Adaptive Regression Splines):** - Explanation: "For MARS, users are encouraged to utilize the default hyperparameters listed in Table s3 as a foundation. In the supplementary file, we provide a comprehensive dataset for readers to model their algorithms. This allows for practical experimentation with the MARS method on our provided data."

3. **MLP-lM, MLP-BR, MLP-SCG (Multi-layer Perceptron):** "The default hyperparameters presented in the table serve as a guide for implementing MLP models. In the supplementary file, readers will find the dataset used in our study. By incorporating these hyperparameters and the provided data, users can model their algorithms, tailoring the architecture to their specific analysis requirements."

4. **RBF (Radial Basis Function):** - Explanation: "Users are encouraged to leverage the default hyperparameters specified in Table s3 when applying the RBF kernel in SVR. To facilitate practical implementation, we have included our dataset in the supplementary file. This allows readers to model their algorithms, fine-tuning parameters based on the provided dataset for optimal results." These additions highlight the practical application of the provided hyperparameters and dataset for readers wishing to model their algorithms.

Table s3. Parameters of the developed models.

| Technique | Hyperparameters (Default Values) |
| --- | --- |
| Adaboost_SVR | SVR (C=1.0, epsilon=0.1, kernel='rbf', gamma='scale') |
| MARS | Default parameters depend on the implementation |
| MLP-lM | MLP Regressor (hidden_layer_sizes= (100,), solver='lbfgs') |
| MLP-BR | MLP Regressor (hidden_layer_sizes= (100,), solver='lbfgs') |
| MLP-SCG | MLP Regressor (hidden_layer_sizes= (100,), solver='scg') |
| RBF | SVR (C=1.0, epsilon=0.1, kernel='rbf', gamma='scale') |
